# Correlation analysis of muscle amino acid deposition and gut microbiota profile of broilers reared at different ambient temperatures

**DOI:** 10.5713/ajas.20.0314

**Published:** 2020-08-30

**Authors:** Yuting Yang, Huan Gao, Xing Li, Zhenhui Cao, Meiquan Li, Jianping Liu, Yingying Qiao, Li Ma, Zhiyong Zhao, Hongbin Pan

**Affiliations:** 1 Yunnan Provincial Key Laboratory of Animal Nutrition and Feed Science, Faculty of Animal Science and Technology, Yunnan Agricultural University, Kunming 650201 China; 2 Department of Animal Husbandry and Veterinary Medicine, College of Agriculture, Kunming University, Kunming 650201, China; 3 Jiangsu Key Laboratory for Molecular and Medical Biotechnology, College of Life Sciences, Nanjing Normal University, Nanjing 210023, China; 4 Yunnan Vocational and Technical College of Agriculture, Kunming 650201, China; 5 Yunnan Animal Science and Veterinary Institute, Kunming 650201, China

**Keywords:** Broiler, Amino Acid Deposition, Fecal Microbiota Composition, Ambient Temperature

## Abstract

**Objective:**

Temperature could influence protein and amino acid deposition as well as gut microbiota profile and composition. However, the specific effects of ambient temperature on amino acids deposition and gut microbiota composition remain insufficiently understood.

**Methods:**

A total of 300 one-day-old Avian broilers were randomly divided into three groups and reared at high, medium, and low temperature (HT, MT, and LT), respectively. Breast muscle and fecal samples were collected for amino acid composition analysis and 16S rRNA gene sequence analysis.

**Results:**

Our data showed that compared to the MT group, there was a decrease of muscle leucine and tyrosine (p<0.05), as well as an increase of methionine in the HT group (p<0.05) and a decrease of serine in the LT group. Examination of microbiota shift revealed that at genus level, the relative abundance of *Turicibacter* and *Parabacteroides* was increased in the HT group (p<0.05) and that the relative abundances of *Pandoraea*, *Achromobacter*, *Prevotella*, *Brevundimonas*, and *Stenotrophomonas* in the LT group were higher than those in the MT group (p<0.05). In addition, there were substantial correlations between microbes and amino acids. In the HT group. *Turicibacter* was negatively correlated with aspartic acid and tyrosine, whereas *Parabacteroides* was positively correlated with methionine (p<0.05). In the LT group, there were multiple positive correlations between *Achromobacter* and arginine, isoleucine or tyrosine; between *Prevotella* and cysteine or phenylalanine; between *Brevundimonas* and cysteine; and between *Stenotrophomonas* and cysteine as well as a negative correlation between *Stenotrophomonas* and serine.

**Conclusion:**

Our findings demonstrated that amino acid content of breast muscle and intestinal microbiota profile was affected by different ambient temperatures. Under heat exposure, augmented abundance of *Parabacteroides* was correlated with elevated methionine. Low temperature treatment may affect muscle tyrosine content through the regulation of *Achromobacter*.

## INTRODUCTION

Environment conditions are crucial to poultry welfare and production, as the animals are highly sensitive to temperature-associated environmental challenges [1]. Long-term exposure of poultry to unfavorable environmental temperatures (high or low) can affect the performance of chickens by reduction of body weight gain, impairment of meat quality, induction of excessive immune responses and elevation of mortality [2]. In addition, heat stress or cold stress lead to prominent changes in the gut microbiota composition [3]. The animal gastrointestinal tract is a major habitat for numerous species of microbes [4], which play various crucial roles including prevention of the colonization of enteric pathogens through the process of competitive exclusion and the production of bacteriostatic and bactericidal substances [5]. Also, the host metabolism, nutrition, health, and growth performance can be influenced by the enteric microbes via modulating energy balance, nutrient utilization and the development of homeostasis [6].

It has been shown that high or low temperature could change broilers’ muscle protein content in broilers [7,8]. A previous study showed that amino acid composition of breast muscle was altered under high ambient temperature [9]. Comparison of germ-free mice and conventionalized mice showed that the latter possessed an altered distribution of free amino acids in the gastrointestinal tract and the gut microbiota affected host amino acid metabolism [10]. However, it remains unclear whether intestinal microorganisms exert different effects on amino acid metabolism in breast muscle under different ambient temperatures over a long-term (day 1 to 42). In light of this, the goal of this study was to examine whether long-term temperature stress plays a role in the correlations between amino acid deposition and gut microbiota composition of broilers.

## MATERIALS AND METHODS

### Animal ethics statement

This study was carried out in accordance with the recommendations of the Institutional Animal Care and Use Committee of Yunnan Agricultural University (approval No.: YNAU 20160016), and complied with the guidelines of the institutional administrative committee and ethics committee of laboratory animals.

### Experimental design and animal management

A total of 300 one-day-old avian chicks were purchased from a local supplier (Hunan Shuncheng Industrial Co. Ltd., Hunan, China) and randomly split into three open-circuit calorimetry chambers that were under high temperature (HT), medium temperature (MT), and low temperature (LT), respectively. The temperatures were achieved in a decremental mode in which they began at 36.5°C (HT), 33.5°C (MT), and 30.5°C (LT), respectively, and were reduced by 0.5°C per day until they reached 22°C, 19°C, and 16°C, respectively (Supplementary Table S1). Chicks in each chamber were further subdivided into three areas and provided with a bedding of rice husks, feed and water *ad libitum*. Apart from the temperature differences, the chickens in all three groups received the same treatments, including diet formulated according to NRC [11] and the same housing (Supplementary Table S2). The chickens were provided with light/dark cycles of 24/0 on D1, 23/1–18/6 from D2 to D8, 12/12 from D9 to D21, 18/6 from D22 to D35, and 18/6–23/1 from D36 to D42 (the light period increased from 18 h on day 36 to 23 h on day 42); the light intensity was 30 to 60 lux for chickens with a weight of less than 160 g (about 1 to 8 d), and 5 to 10 lux for chickens with a weight above 160 g (about 9 to 42 d). The relative humidity was 30% to 50% from D1 to D7, 40% to 60% from D8 to D21, and 50% to 70% from D22 to D42.

### Sample collection

On day 42, twelve birds (six male and six female) from each group were randomly selected and sacrificed by cervical dislocation. Feces were sampled, preserved in liquid nitrogen, and subjected to DNA extraction and polymerase chain reaction (PCR) amplification. Both sides of the breast muscle were removed and preserved at −20°C for amino acid analysis.

### Analysis of muscular amino acids

For amino acid analysis, the samples preparation was as follows: each breast muscle sample weighted 30 mg was transferred into a hydrolysis tube and digested in 10 mL of 6 N HCl at 110°C for 24 h. After filtering, 0.3 mL of the mixture was transferred into a tube for drying in an oven at 140°C for 1 h. Afterwards, 3 mL sodium citrate buffer solution (pH 2.2) was used to dissolve the desiccated filtrate sample, followed by the determination of the amino acid composition and content of breast muscle using an amino acid analyzer (Sykam S-433D, Munich, Germany).

### DNA extraction and polymerase chain reaction amplification

Genomic DNA was extracted from fecal samples using the QIAamp Fast DNA Stool Mini Kit (Qiagen, Cat No.19593, Dusseldorf, Germany) according to manufacturer’s protocols. The DNA samples were used as the template for PCR. The hypervariable V3-V4 region of the bacteria 16S ribosomal RNA genes were amplified using the primer pair 341 F 5′-CCTACGGGRSGCAGCAG)-3′ and 806 R 5′-GGAC TACVVGGGTATCTAATC-3′ with the following cycling condition: a denaturing step at 95°C for 3 min, followed by 30 cycles at 98°C for 20 s, 58°C for 15 s, and 72°C for 20 s and a final extension at 72°C for 5 min. The PCR reactions were performed in 30 μL mixture containing 15 μL of 2×KAPA Library Amplification Ready Mix, 1 μL of each primer solution (10 μM) and 50 ng of template DNA.

### Illumina HiSeq PE250 sequencing

Amplicons were electrophoresed in 2% agarose gels, followed by extraction from the gel, purification using the AxyPrep DNA Gel Extraction Kit (Axygen Biosciences, Union City, CA, USA) according to the manufacturer’s instructions and quantified using Qubit 2.0 (Invitrogen, Carlsbad, CA, USA). Libraries were prepared and sequenced on a HiSeq platform (Illumina, Inc., San Diego, CA, USA) for paired-end reads of 250 bp, which were overlapping on their 3 ends for concatenation into longer contigs. DNA extraction, library construction and sequencing were performed at Realbio Genomics Institute (Realbio, Shanghai, China). Processing of sequence data was made as previously described [12].

### Statistical analysis

Experimental data including growth performance, amino acid composition of breast muscle and microbial abundances were analyzed using the SPSS 22.0 software (IBM SPSS Statistics for Windows; NY: IBM Corp, Armonk, NY, USA). Shapiro-Wilk test was applied to assess normality. After logarithmic transformation, only data of growth performance and amino acid composition of breast muscle displayed a normal distribution. The general linear model analysis with Duncan multiple comparison test was used for parametric data and Kruskal-Wallis analysis of variance performed on ranks was used for the microbial abundances at phylum and genus level. The growth performance and amino acid data were expressed as the mean, and the pooled standard error of mean was provided. To assess the correlation between dominant genera and amino acids, the Spearman’s test in GraphPad Prism 7.0 was performed, and p<0.05 was considered as the criterion for statistical significance.

## RESULTS

### Growth performance

The three temperature treatments resulted in detectable differences in the growth performance of the broilers. On day 42, the final body weight, average daily gain and breast muscle weight of broilers in the HT and MT groups were significantly higher (p<0.05) than that in the LT group. The average daily feed intake was 98.31, 97.03, and 91.1 for HT, MT, and LT groups, respectively. The ratio of feed gain of broilers in the LT (2.18) group was higher than that in the HT (1.98) group (p<0.05) and both groups were higher than that in the MT (1.87) group (p<0.05) (Table 1).

### Amino acid deposition and crude protein analysis

We next examined the influences of the three temperature treatments on amino acid deposition and crude protein (CP) content in the breast muscle. As shown in Table 2, CP content in the LT group was higher than that in the MT and HT groups (p<0.05). The content of serine in breast muscle of MT group was significantly higher (p<0.05) than that of LT group. Lower methionine of breast muscle in MT group was observed compared with the HT and LT groups (p<0.05). Compared with the MT group, the broilers in the HT group had less leucine (p<0.05), and the HT chicks had lower tyrosine than the MT and LT chicks (p<0.05).

### Sequence and data analysis

**Figure 1:**
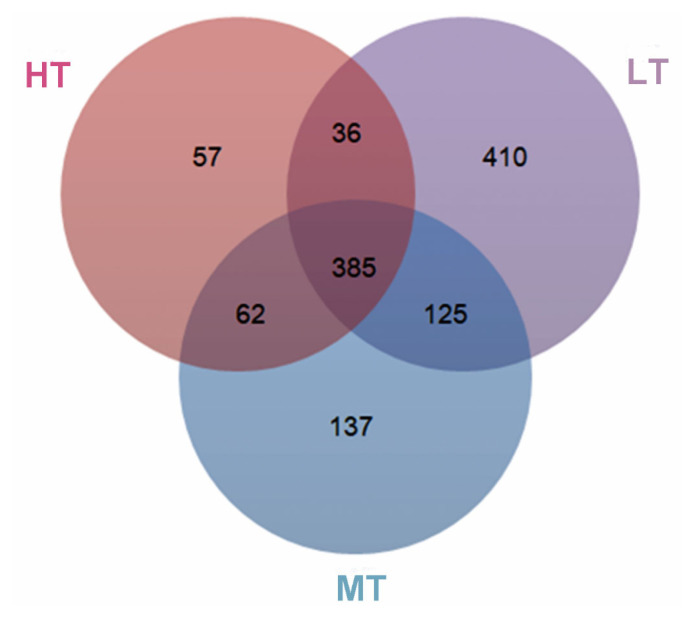
Venn diagram of OTUs clustered at 97% sequence identity across HT, MT, and LT groups. The different colors respectively represent HT, MT, LT groups, and the overlapping area represent shared OTUs numbers between different groups. OUTs, operational taxonomic unit; HT, high temperature; MT, medium temperature; LT, low temperature.

Analysis of 16S rRNA gene amplicons generated 2,124,545 clean reads from the 36 fecal samples, resulting in 37,479 to 64,768 reads per sample with an average length of 416 bp. To avoid the effect of the sequence depth on the measurement of microbial composition, we rarefied the library size to 32,296 reads per sample using the rarefy function in QIIME pipeline (Supplementary Table S3). With a 97% sequence similarity, 385 core operational taxonomic units (OTUs) were generated. As a consequence, and the OTUs numbers were 540, 709, or 956 for the HT, MT, or LT groups, and the proportion of core taxa in the feces of broilers was 71%, 54%, or 40%, respectively (Figure 1).

**Figure 2:**
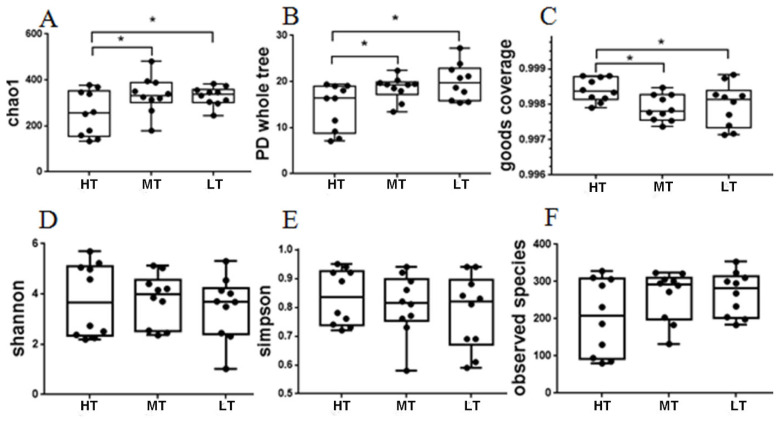
Diversity estimation of the16S rRNA gene libraries of the broilers’ feces. The boxes represent interquartile range (IQR), and the line between boxes indicate median value. * p<0.05. HT, high temperature; MT, medium temperature; LT, low temperature.

The microbial diversity indices were calculated based on the OTUs of each library. And the Chao1 index, phylogenetic diversity (PD) whole tree index, good’s coverage, Shannon index, observed species index, and Simpson index (Supplementary Table S4). The results of alpha diversity analysis is shown in Figure 2, showing that the indices of chao1 and PD whole tree in the MT and LT groups were significantly higher (p<0.05) than those in the HT group, and that the index of good’s coverage in the HT group was significantly higher (p<0.05) than those in the MT and LT groups.

### Comparison of gut bacterial composition at phylum level based on 16S rRNA amplicon sequence

**Figure 3:**
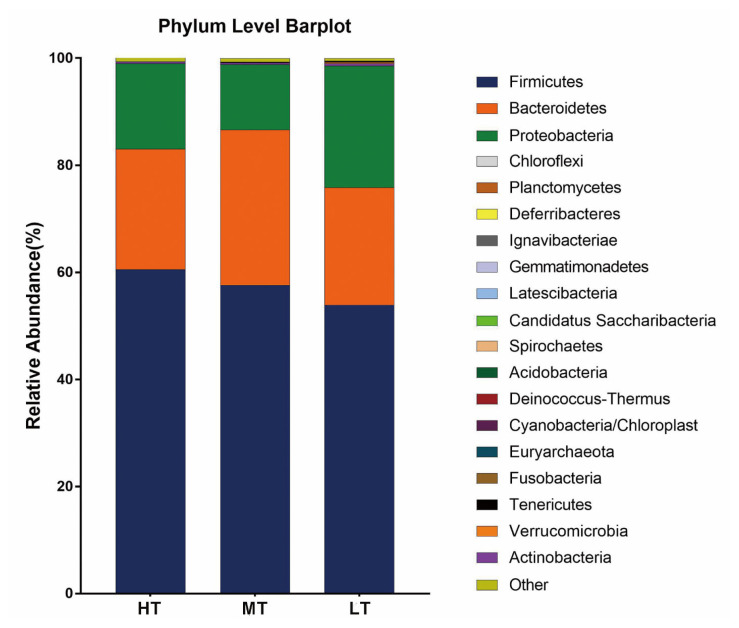
Composition of the dominant microbiome at phylum level. The composition of each sample is based on the ribosomal database project (RDP) taxonomic assignment of the 16S rDNA sequences. HT, high temperature; MT, medium temperature; LT, low temperature.

There were 8, 16, and 14 phyla being identified within the complete dataset in the HT, MT, and LT groups, respectively (Supplementary Figure S1). Firmicutes, Bacteroidetes and Proteobacteria were the only three phyla shared among the three groups, with each accounting for ≥10% of the fecal microbiota. Overall, the relative abundance of the three major phyla was comparable among the three groups. Specifically, the proportions of Firmicutes were 60.56% in the HT group, 57.62% in the MT group and 53.92% in the LT group; that of Bacteroidetes were 22.48% in the HT group, 28.98% in the MT group and 21.91% in the LT group; that of Proteobacteria were 15.95% in the HT group, 12.22% in the MT group and 22.76% in the LT group, respectively (Figure 3). The other bacterial phyla had relative abundances lower than 1% in all groups at varying magnitudes.

### Comparison of gut bacterial composition at genus level based on 16S rRNA amplicon sequence

At the genus level, the top 20 abundant genera for all three groups were *ClostridiumXI*, *Bacteroides*, *Escherichia/Shigella*, *Barnesiella*, *Enterococcus*, *Lactobacillus*, *Pandoraea*, *Alistipes*, *Turicibacter*, *Clostridium XIVa*, *Faecalibacterium*, *Achromobacter*, *Butyricicoccus*, *Oscillibacter*, *Variovorax*, *Clostridium IV*, *Clostridium XIVb*, *Clostridium* sensu strict, *Phascolarctobacterium*, and *Parabacteroides* in the three groups. Among these, the major taxa included *Clostridium XI* (8.77% in HT, 17.76% in MT, and 25.68% in LT), *Bacteroides* (11.29% in HT, 11.24% in MT, and 13.06% in LT), *Escherichia/Shigella* (15.84% in HT, 11.36% in MT, and 8.31% in LT) and *Barnesiella* (7.82% in HT, 15.80% in MT, and 5.07% in LT) (Supplementary Figure S2).

**Figure 4:**
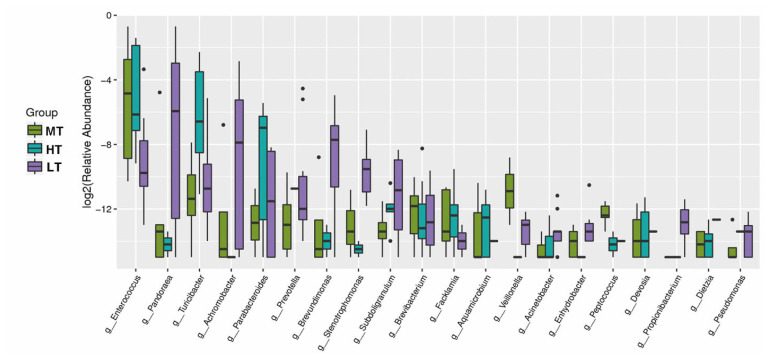
Boxplot of the top 20 most differentially abundant genus in the HT, MT and LT groups. The value of log_2_ (relative abundance) was used in the plot. The dots in the figure represent deviation values. HT, high temperature; MT, medium temperature; LT, low temperature.

The relative abundances of *Turicibacter* (4.88%), *Parabacteroides* (0.73%), *Brevibacterium* (0.043%), *Facklamia* (0.028%), *Aquamicrobium* (0.016%) or *Dietzia* (0.0021%) were higher in the HT group compared with that in the MT group (p<0.05). In addition, the relative abundance of *Enterococcus* in the MT (13%) and HT (12%) groups was higher than that in the LT (1%) group (p<0.05); *Veillonella* in MT group was higher than in HT and LT groups (p<0.05). The abundance of *Pandoraea* (9.9%), *Achromobacter* (2.14%), *Prevotella* (0.61%), *Brevundimonas* (0.55%), *Stenotrophomonas* (0.12%), *Subdoligranulum* (0.092%), *Enhydrobacter* (0.011%), *Acinetobacter* (0.01%), and *Propionibacterium* (0.011%) in the LT group were significantly higher than those in HT and MT groups (p<0.05) (Figure 4). The results indicated that the different temperature treatments led to substantial shift of gut microbiota.

### Correlations between microbes and amino acid deposition

**Figure 5:**
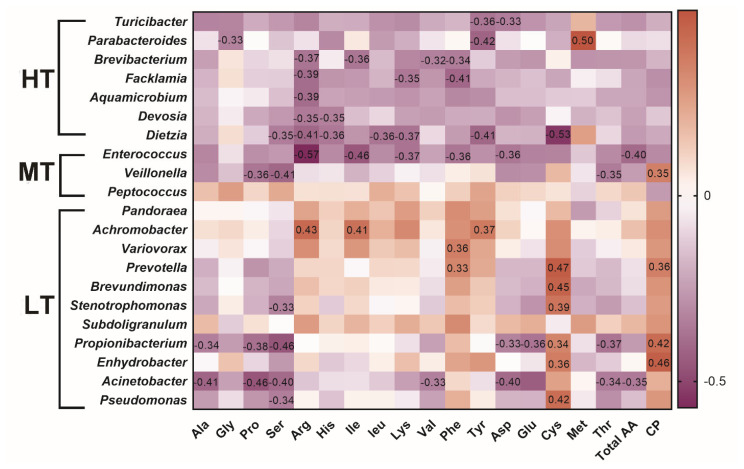
Heatmap of the correlation between microbial changes and amino acids. Spearman’s test was used to calculate correlation coefficient. The negative correlation was expressed by purple color, and the positive correlation was expressed by red color. HT, high temperature; MT, medium temperature; LT, low temperature.

As shown in Figure 5, positive correlation was revealed between the protein content and *Veillonella*. In the HT group, *Turicibacter* was negatively correlated with aspartic acid (p = 0.044, R = −0.33) and tyrosine (p = 0.030, R = −0.36); *Parabacteroides* was negatively associated with glycine or tyrosine and positively correlated with methionine (p = 0.0019, R = 0.50); *Dietzia* was negatively correlated with leucine (p = 0.030, R = −0.36). In the MT group, negative correlation were detected between *Enterococcus* and arginine (p = 0.00028, R = −0.57) or isoleucine (p = 0.0051, R = −0.46) and between *Veillonella* and serine (p = 0.013, R = −0.40), threonine (p = 0.038, R = −0.35), or proline (p = 0.030, R = −0.36). In the LT group, associations were identified between *Achromobacter* and arginine (p = 0.0083, R = 0.43), isoleucine (p = 0.014, R = 0.41), between *Variovorax* and phenylalanine (p = 0.03, R = 0.36), between *Prevotella* and cysteine (p = 0.0034, R = 0.47), or phenylalanine (p = 0.046, R = 0.33), between *Brevundimonas*, and cysteine (p = 0.0053, R = 0.45), between *Stenotrophomonas* and serine (p = 0.048, R = −0.33), or cysteine (p = 0.018, R = 0.39) (Supplementary Table S5, Table S6). The results indicated that gut microbiota composition influences amino acid composition.

## DISCUSSION

Environment is important factor affecting the performance of modern poultry production [13]. Birds are homeotherms and can maintain their body temperature within a narrow range. However, when the body temperature substantially deviates from the normal range resulting from exposure to unfavorable environmental conditions, a cascade of irreversible thermoregulatory events may occur, which are detrimental and even lethal to the birds [14]. Studies have shown that long-term exposure to cold environment leads to a decrease in protein synthesis in rats [15] and calves [16]. Differences in composition of amino acids, especially those essential amino acids, can greatly influence the protein synthesis. A previous study reported that after 14 days (28 d to 42 d) of heat exposure (environment temperature was 32°C), muscle amino acid concentration was reduced of broilers [17]. The detrimental effect on muscular amino acids was also observed in rats exposed to cold stress at 4°C [18]. These results are consistent with our results to collectively show that heat and cold stresses influence the content of muscular crude protein and amino acid composition.

In the present study, the contents of serine and glycine were significantly decreased by long-term cold stress, as were those of leucine, histidine and tyrosine by heat treatment in broilers. The methionine levels in HT and LT groups were significantly higher than that in MT group. Studies have revealed that exposure to extreme temperatures led to oxidative stress [19]. As an essential amino acid in chickens, methionine has a positive effect on the expression of stress related genes and thus helps to protect cells from oxidative challenge [20]. It is plausible that under extreme environment, the broilers require a higher methionine concentration. Therefore, temperature is one of the main factors affecting amino acid metabolism in broilers.

In healthy chickens, the composition of intestinal microbiota remains relatively stable, which may be disrupted by various factors such as pathogen invasion, antibiotic administration and environmental stress (e.g., extremely high or low temperature, overcrowding, poor feeding, transportation) [21]. Fecal microbiome analyses suggested bacterial richness was a major marker for gut health [22]. And Chao1 indices are usually used to estimate bacterial richness. In this study, Chao1 showed that the gut microbiota in MT and LT groups were more abundant than that in HT group.

It has been shown that intestinal microflora could be considered as an important indicator of intestinal health [23]. The composition and relative abundance of intestinal microbiota are influenced by environmental temperature [24]. *Enterococcus* is a large genus of lactic acid bacteria in the phylum Firmicutes [25]. Although some species of *Enterococcus* are potentially pathogenic, their toxicity is generally low and they are natural commensals in human and animal gastrointestinal tracts [26]. Our study showed that the proportion of *Enterococcus* in the MT and HT groups was higher than that in the LT group, indicating their abundance was affected by heat stress. *Barnesiella* is a genus in the family of Porphyromonadaceae and is a major genus detected in mouse intestines. It is associated with hyperglycemia, insulin resistance, hepatic steatosis and inflammation in rodents [27]. *Barnesiella* is also one of the most abundant genera in human feces and can be used to treat vancomycin-resistant Streptococcus faecalis colonization [28]. In this study, *Barnesiella* was a predominant in all samples and its relative abundance in the MT group was higher than that of the other two groups. The results indicated *Barnesiella* was also one of the most abundant genera in broilers and its abundance was influenced by both heat and cold stresses.

The resident species of the gut microbiota can alter the bioavailability of amino acids by utilizing of several amino acids that are originated from both alimentary and endogenous proteins [29]. Additionally, the gut microbiota can synthesize amino acids and provide them to the host [30]. Therefore, the gut microbiota is a potential regulatory factor in amino acid homeostasis which may affect the muscular amino acids deposition. In the present study, *Parabacteroides* was augmented in the feces of the HT-treated broilers. The correlation analysis revealed that in the HT group, this genus was negatively associated with glycine or tyrosine and positively correlated with methionine. The results suggested that under heat exposure, augmenting the abundance of *Parabacteroides* correlated with elevated concentration of methionine. In addition, *Achromobacter* was enriched in the feces of the LT group, and *Achromobacter* was positively correlated with tyrosine in the MT and LT groups, suggesting that low temperature treatment may affect tyrosine through the regulation of *Achromobacter*. Moreover, our analyses revealed that *Stenotrophomonas* was negatively correlated with serine and that the serine content in the LT group was lower than that in the MT group, suggesting that low temperature treatment may affect serine metabolism and that it might involve *Stenotrophomonas*.

16S rRNA gene-based analysis revealed that whereas the proportion of the three major phyla, namely Firmicutes, Bacteroidetes and Proteobacteria, were comparable among the three groups, there were substantial differences at genus level. Specifically, significant differences of multiple genera (eg., *Turicibacter*, *Parabacteroides*, *Enterococcus*, *Pandoraea*, *Achromobacter*, *Variovorax*, *Prevotella*, *Brevundimonas*, and *Stenotrophomonas*) were found in all three groups. Furthermore, our findings suggested some connections between gut microbiota and muscular amino acid composition. Our findings provide new evidence for better understanding the involvement of gut microbiota in regulation of amino acid metabolism and how the process is affected by ambient temperatures. Furthermore, our study also provides crucial experimental basis for developing microbiota-based supplements to improve meat quality in broilers.

## Figures and Tables

**Table 1:** Growth performance of broilers in different environmental temperatures

Group ID	HT1)	MT1)	LT1)	SEM	p-value
FBW (g)	2,141.50^a^	2,225.00^a^	1,801.00^b^	43.16	<0.001
ADFI (g)	98.31	97.03	91.1	1.49	0.108
ADG (g)	49.89^a^	51.89^a^	41.79^b^	1.03	<0.001
F/G	1.98^b^	1.87^c^	2.18^a^	0.023	<0.001
Breast muscle weight (g)	374.50^a^	386.50^a^	321.50^b^	9.56	0.008

Values reported as means (n = 12).

SEM, standard error of means for 12 broilers each; FBW, final body weight; ADFI, average daily feed intake; ADG, average daily gain; F/G, the ratio of feed gain.

1) HT, high temperature; MT, medium temperature; LT, low temperature.

a,b Means in the same row with different superscripts differ statistically (p<0.05).

**Table 2:** Effects of different environmental temperatures on crude protein and amino acid composition in broilers breast muscle

Group ID	HT1)	MT1)	LT1)	SEM	p-value
Alanine	1.34	1.37	1.32	0.013	0.602
Glycine	1.04^ab^	1.12^a^	1.04^b^	0.012	0.061
Proline	0.85	0.86	0.82	0.0082	0.446
Serine	0.70^ab^	0.8^a^	0.67^b^	0.0088	0.027
Arginine	1.53	1.6	1.65	0.016	0.205
Histidine	0.70^b^	0.78^a^	0.75^ab^	0.016	0.052
Isoleucine	1.25	1.25	1.38	0.011	0.27
Leucine	2.14^b^	2.26^a^	2.24^ab^	0.019	0.039
Lysine	2.33	2.45	2.47	0.024	0.307
Valine	1.27	1.28	1.24	0.013	0.737
Phenylalanine	1.18	1.21	1.27	0.011	0.149
Tyrosine	0.85^b^	0.95^a^	0.91^a^	0.0095	0.001
Aspartic acid	2.15	2.21	2.17	0.21	0.661
Glutamic acid	3.47	3.53	3.42	0.034	0.661
Cysteine	0.23	0.28	0.27	0.0037	0.284
Methionine	0.70^a^	0.47^b^	0.62^a^	0.011	0.001
Threonine	0.85	0.86	0.83	0.011	0.776
Total AA	22.62	23.28	22.99	0.22	0.594
Crude protein	23.91^b^	24.07^b^	25.69^a^	0.23	<0.001

Values reported as means (n = 12).

SEM, standard error of means for 12 broilers each; AA, amino acid.

1) HT, high temperature; MT, medium temperature; LT, low temperature.

a,b Means in the same row with different superscripts differ statistically (p<0.05).
