# Supplementary material for: Correlation analysis of muscle amino acid deposition and gut microbiota profile of broilers reared at different ambient temperatures
Source: Anim Biosci. 2020 Aug 30;34(1):93–101. doi: 10.5713/ajas.20.0314 (PMC7888499; doi:10.5713/ajas.20.0314)
Supplement: Supplementary file 1 [file ajas-20-0314-suppl.pdf]

SUPPLEMENTARY INFORMATION

Table S1: Temperature management.

Day	HF group	MF group	LF group
1	36.5	33.5	30.5
2	36	33	30
3	35.5	32.5	29.5
4	35	32	29
5	34.5	31.5	28.5
6	34	31	28
7	33.5	30.5	27.5
8	33	30	27
9	32.5	29.5	26.5
10	32	29	26
11	32	29	26
12	31.5	28.5	25.5
13	31	28	25
14	31	28	25
15	30.5	27.5	24.5
16	30.5	27.5	24.5
17	30	27	24
18	30	27	24
19	29.5	26.5	24.5
20	29	26	23
21	29	26	23
22	28.5	25.5	22.5
23	28	25	22
24	28	25	22
25	27.5	24.5	21.5
26	27.5	24.5	21.5
27	27	24	21
28	27	24	21
29	26.5	23.5	20.5
30	26	23	20
31	26	23	20
32	25.5	22.5	19.5
33	25	22	19
34	25	22	19
35	24.5	21.5	18.5
36	24	21	18
37	24	21	18
38	23.5	20.5	17.5
39	23	20	17
40	23	20	17
41	22.5	19.5	16.5
42	22	19	16

When the temperature is 0.5 degree lower than the expected temperature, the heater is turned on and the fan is closed. When the heater is 0.5 degree higher, the heater is closed and the ventilator opens.

Table S2: Ingredient and nutrient composition (%, as feed) of the experimental diets.

Ingredient (%)	Starter diet (%) 1 to 21d	Finisher diet (%) 22 to 42 d
corn（first）	60.00	66.40
Soybean meal 43	27.40	20.00
corn protein flour	6.00	8.00
Imported fish meal	1.00	0.00
Soybean oil	1.90	1.60
Stone powder	1.10	1.20
Calcium hydrogen phosphate	1.10	1.14
salt	0.35	0.35
Met	0.07	0.06
Lys	0.08	0.25
1% broiler premix	1.00	1.00
Calculated nutrients level (%)		
ME（Mcal/kg）	2950	3000
CP	21.00	19.00
Ca	0.81	0.80
P	0.50	0.50
Lys	1.10	1.00
Met	0.50	0.38
Met + Cys	0.90	0.72
Thr	0.80	0.74
Try	0.20	0.18

ME: Metabolizable energy, CP: Crude protein, Lys: lysine, Met: Methionine, Cys: Cystine, Ca: Calcium, P: Phosphorus, Thr: Threonine, Try: Tryptophan.

Table S3: Description of the assembly results of fecal microbiota from chicken Broiler.

Sample name	Clean Reads	Mapped reads	Mapped ratio(%)	Bases(bp)	Q20(%)	GC(%)	Average length(bp)	OTUs
HF-1	57229	55938	97.7441507	24316291	95.33%	52.80%	424	213
HF-2	60348	56162	93.0635647	25441189	95.12%	53.49%	421	113
HF-3	63063	60040	95.2063809	26752715	95.31%	53.23%	424	131
HF-4	63143	60918	96.4762523	26408619	95.48%	52.79%	418	102
HF-5	61121	57590	94.2229348	25948347	95.23%	53.20%	424	87
HF-6	60312	54833	90.9155724	25189154	95.27%	51.62%	417	321
HF-7	58056	49323	84.9576271	24122611	94.92%	50.81%	415	342
HF-8	56226	48675	86.57027	23236405	95.32%	51.25%	413	351
HF-9	64622	62768	97.131008	27549784	95.32%	51.26%	426	175
HF-10	64768	58113	89.7248641	26883001	95.49%	51.68%	415	339
HF-11	61282	54027	88.1612872	25396973	95.35%	51.73%	414	266
HF-12	63143	47971	75.9720001	26068943	91.72%	52.64%	412	376
MF-1	61932	55344	89.3625266	25852679	95.24%	52.41%	417	306
MF-2	60639	53160	87.6663533	25349383	95.14%	51.68%	418	355
MF-3	54021	52024	96.3032895	23018797	95.16%	52.88%	426	168
MF-4	57213	53690	93.8423086	23933030	94.29%	52.07%	418	333
MF-5	62620	59270	94.6502715	25961389	95.51%	52.69%	414	128
MF-6	64044	58329	91.0764474	26340549	95.90%	52.24%	411	313
MF-7	63499	53085	83.5997417	26533098	92.92%	52.53%	417	362
MF-8	37479	34618	92.3663919	15591324	94.36%	52.53%	416	379
MF-9	61244	57732	94.2655607	25056626	95.71%	53.21%	409	227
MF-10	48669	46473	95.4878876	20188152	95.29%	52.98%	414	344
MF-11	55063	52862	96.0027605	23375888	95.14%	52.09%	424	242
MF-12	64047	56066	87.5388387	26416592	95.26%	52.58%	412	354
LF-1	57631	51524	89.4032725	23690755	95.78%	51.65%	411	342
LF-2	63203	58144	91.9956331	26046261	95.44%	51.61%	412	318
LF-3	55177	54377	98.5501205	22260059	96.57%	53.10%	403	257
LF-4	57821	55081	95.2612373	23674067	95.89%	50.98%	409	368
LF-5	55508	53562	96.494199	22746502	95.19%	53.45%	409	234
LF-6	64250	61455	95.6498055	26370649	95.84%	52.96%	410	513
LF-7	54829	53414	97.4192489	22204550	95.94%	53.27%	404	234
LF-8	56336	53988	95.83215	23977622	94.45%	53.60%	425	86
LF-9	60283	54942	91.1401224	25028373	95.15%	50.30%	415	252
LF-10	56196	54277	96.5851662	23631671	94.54%	51.21%	420	323
LF-11	63483	60273	94.9435282	26709185	94.95%	51.78%	420	331
LF-12	56045	51245	91.4354537	23550718	95.04%	53.42%	420	344

Table S4: Statistical analysis of bacterial alpha diversity in the samples.

Alpha name	chao1	Observed species	PD whole tree	shannon	simpson	Goods coverage
HF-1	251.8077	186	16.37256	2.729092	0.778543	0.998033
HF-2	133.4615	85	7.80973	2.511034	0.736755	0.9988
HF-3	141.5714	94	9.17244	2.187594	0.715671	0.998767
HF-4	158.75	80	7.62351	2.37364	0.75884	0.9988
HF-5	122	64	7.05308	2.25208	0.733048	0.999033
HF-6	338.5	289	18.0648	4.584943	0.891813	0.998167
HF-7	346.0294	310	19.35494	5.21685	0.924309	0.998333
HF-8	368.8857	328	19.08529	5.705228	0.950089	0.9982
HF-9	179.0435	130	11.53518	1.258219	0.304391	0.9984
HF-10	377.7241	306	18.99568	4.981735	0.922444	0.997833
HF-11	260.2857	231	16.3588	5.059324	0.945591	0.998633
HF-12	400.25	346	20.23938	5.694349	0.944359	0.9979
MF-1	325.3333	272	17.94219	4.146771	0.892648	0.997833
MF-2	394	321	19.70437	4.398385	0.859757	0.997533
MF-3	179.3571	132	13.44918	2.446185	0.732617	0.998267
MF-4	339.2	295	20.23233	3.846655	0.768098	0.998267
MF-5	174.25	88	9.99403	2.359785	0.755974	0.998467
MF-6	319.875	289	18.51471	5.598725	0.954983	0.9987
MF-7	388.0294	323	19.28001	4.200025	0.812867	0.997767
MF-8	429.62	368	24.71251	5.029593	0.924521	0.997367
MF-9	314.4	183	15.11882	2.321112	0.575816	0.997567
MF-10	480.6667	301	22.36378	3.703119	0.823089	0.9967
MF-11	266.2778	203	19.37017	2.535721	0.627914	0.997733
MF-12	350.2941	305	19.46692	5.119045	0.938738	0.998133
LF-1	383.3125	310	20.75772	5.29659	0.939276	0.9977
LF-2	346.037	295	18.67032	5.326801	0.939082	0.998233
LF-3	304.1	204	17.7466	0.907081	0.179359	0.9974
LF-4	374.303	353	27.25716	4.017897	0.69477	0.998733
LF-5	245.3571	198	15.84687	2.435011	0.60787	0.998267
LF-6	520.8971	451	32.55745	3.492315	0.693468	0.996733
LF-7	297.2188	183	15.55139	1.020899	0.237733	0.997133
LF-8	122	83	9.06309	2.320504	0.590193	0.999567
LF-9	311.7143	233	15.39086	4.137256	0.878445	0.998067
LF-10	355.0732	268	22.55042	3.681013	0.842476	0.997167
LF-11	331.8	300	21.14476	3.704149	0.812076	0.9982
LF-12	345.8846	323	23.83526	4.544589	0.826577	0.998833

Table S5: The *P*-value between amino acids and bacterial.

	Ala	Gly	Pro	Ser	Arg	His	Ile	leu	Lys	Val	Phe	Tyr	ASP	Glu	Cys	Met	Thr	CP	Total AA
Turicibacter	0.06	0.06	0.23	0.12	0.06	0.24	0.22	0.09	0.08	0.22	0.29	0.03	0.04	0.10	0.18	0.26	0.13	0.24	0.09
Parabacteroides	0.69	0.05	0.95	0.40	0.69	0.07	0.75	0.13	0.24	0.71	0.92	0.01	0.69	0.98	0.25	0.00	0.95	0.66	0.61
Brevibacterium	0.15	0.72	0.55	0.67	0.03	0.78	0.03	0.33	0.06	0.05	0.04	0.49	0.14	0.11	0.83	0.10	0.11	0.28	0.11
Facklamia	0.28	0.66	0.50	0.48	0.02	0.11	0.12	0.28	0.03	0.11	0.01	0.21	0.28	0.38	0.19	0.82	0.65	0.09	0.19
Aquamicrobium	0.34	0.87	0.76	0.37	0.02	0.18	0.15	0.13	0.10	0.18	0.07	0.08	0.36	0.36	0.45	0.48	0.20	0.11	0.20
Devosia	0.29	0.79	0.21	0.12	0.04	0.05	0.35	0.57	0.16	0.14	0.22	0.23	0.10	0.15	0.87	0.26	0.39	0.43	0.17
Dietzia	0.24	0.64	0.50	0.04	0.01	0.03	0.11	0.03	0.03	0.60	0.14	0.01	0.28	0.27	0.00	0.13	0.57	0.21	0.13
Enterococcus	0.06	0.67	0.09	0.12	0.00	0.07	0.01	0.08	0.03	0.18	0.03	0.09	0.03	0.05	0.06	0.44	0.07	0.07	0.02
Veillonella	0.07	0.37	0.03	0.01	0.63	0.72	0.27	0.52	0.84	0.68	0.81	0.68	0.08	0.09	0.29	0.71	0.04	0.03	0.14
Peptococcus	0.34	0.12	0.55	0.18	0.66	0.66	0.68	0.21	0.36	0.93	0.48	0.15	0.51	0.59	0.70	0.53	0.59	0.13	0.40
Pandoraea	0.90	0.90	0.90	0.72	0.14	0.47	0.22	0.39	0.11	0.50	0.06	0.13	0.67	0.92	0.29	0.13	0.53	0.11	0.70
Achromobacter	0.65	0.59	0.80	0.57	0.01	0.55	0.01	0.22	0.05	0.73	0.07	0.02	0.52	0.93	0.07	0.88	0.81	0.06	0.34
Variovorax	0.79	0.72	0.90	0.51	0.07	0.59	0.09	0.42	0.31	0.84	0.03	0.17	0.81	0.69	0.06	0.50	0.81	0.09	0.76
Prevotella	0.25	0.85	0.10	0.22	0.55	0.56	0.91	0.57	0.59	0.77	0.05	0.19	0.30	0.30	0.00	0.45	0.33	0.03	0.67
Brevundimonas	0.33	0.96	0.29	0.20	0.34	0.56	0.50	0.76	0.77	0.63	0.13	0.41	0.33	0.26	0.01	0.20	0.32	0.08	0.63
Stenotrophomonas	0.21	0.80	0.26	0.05	0.53	0.48	0.59	0.90	0.90	0.47	0.32	0.46	0.27	0.10	0.02	0.26	0.14	0.11	0.38
Subdoligranulum	0.30	0.48	0.67	0.97	0.11	0.55	0.26	0.49	0.20	0.40	0.06	0.62	0.30	0.21	0.78	0.11	0.49	0.09	0.28
Propionibacterium	0.04	0.14	0.02	0.00	0.99	0.85	0.82	0.95	0.68	0.16	0.33	0.94	0.05	0.03	0.04	0.68	0.03	0.01	0.13
Enhydrobacter	0.91	0.36	0.57	0.12	0.59	0.44	0.58	0.82	0.34	0.80	0.15	0.09	0.98	0.73	0.03	0.30	0.48	0.00	0.93
Acinetobacter	0.01	0.15	0.00	0.02	0.42	0.64	0.65	0.45	0.15	0.05	0.58	0.65	0.02	0.01	0.14	0.90	0.04	0.21	0.04
Pseudomonas	0.14	0.63	0.11	0.04	0.88	0.40	0.86	0.85	0.77	0.17	0.22	0.78	0.28	0.27	0.01	0.76	0.09	0.09	0.35

$P < 0.05$ is marked with blue color, $P < 0.01$ is marked with red color.

Table S6: The R-value between amino acids and bacterial.

	Ala	Gly	Pro	Ser	Arg	His	Ile	leu	Lys	Val	Phe	Tyr	ASP	Glu	Cys	Met	Thr	CP	Total AA
Turicibacter	-0.32	-0.32	-0.21	-0.26	-0.32	-0.20	-0.21	-0.29	-0.30	-0.21	-0.18	-0.36	-0.34	-0.28	-0.23	0.19	-0.26	-0.20	-0.28
Parabacteroides	-0.07	-0.33	-0.01	-0.14	-0.07	-0.31	0.06	-0.26	-0.20	-0.06	0.02	-0.42	-0.07	0.00	-0.20	0.50	0.01	-0.08	-0.09
Brevibacterium	-0.24	0.06	-0.10	-0.07	-0.37	-0.05	-0.36	-0.17	-0.31	-0.33	-0.34	-0.12	-0.25	-0.27	0.04	-0.28	-0.27	-0.18	-0.27
Facklamia	-0.19	0.08	-0.12	-0.12	-0.39	-0.27	-0.26	-0.18	-0.35	-0.27	-0.41	-0.21	-0.18	-0.15	-0.22	-0.04	-0.08	-0.29	-0.22
Aquamicrobium	-0.16	-0.03	-0.05	-0.15	-0.39	-0.23	-0.24	-0.26	-0.28	-0.23	-0.31	-0.29	-0.16	-0.16	-0.13	-0.12	-0.22	-0.27	-0.22
Devosia	-0.18	-0.05	-0.22	-0.26	-0.35	-0.33	-0.16	-0.10	-0.24	-0.25	-0.21	-0.21	-0.28	-0.25	-0.03	-0.19	-0.15	-0.13	-0.24
Dietzia	-0.20	0.08	-0.12	-0.34	-0.41	-0.36	-0.27	-0.36	-0.37	-0.09	-0.25	-0.41	-0.18	-0.19	-0.53	0.26	-0.10	-0.21	-0.26
Enterococcus	-0.32	-0.07	-0.29	-0.27	-0.57	-0.30	-0.46	-0.30	-0.37	-0.23	-0.36	-0.29	-0.36	-0.33	-0.32	-0.13	-0.31	-0.30	-0.40
Veillonella	-0.30	-0.15	-0.36	-0.41	-0.08	-0.06	-0.19	-0.11	-0.03	-0.07	0.04	0.07	-0.30	-0.29	0.18	-0.06	-0.35	0.35	-0.25
Peptococcus	0.16	0.27	0.10	0.23	0.08	0.08	0.07	0.22	0.16	0.02	0.12	0.25	0.11	0.09	0.07	-0.11	0.09	-0.25	0.15
Pandoraea	0.02	0.02	-0.02	-0.06	0.25	0.12	0.21	0.15	0.27	0.12	0.31	0.26	0.07	-0.02	0.18	-0.26	-0.11	0.27	0.07
Achromobacter	0.08	0.09	0.04	-0.10	0.43	0.10	0.41	0.21	0.33	0.06	0.31	0.37	0.11	0.01	0.31	0.03	0.04	0.31	0.16
Variovorax	-0.05	0.06	-0.02	-0.11	0.31	0.09	0.28	0.14	0.17	-0.04	0.36	0.23	0.04	-0.07	0.31	-0.12	-0.04	0.29	0.05
Prevotella	-0.20	-0.03	-0.28	-0.21	0.10	0.10	-0.02	0.10	0.09	-0.05	0.33	0.22	-0.18	-0.18	0.47	-0.13	-0.17	0.36	-0.07
Brevundimonas	-0.17	-0.01	-0.18	-0.22	0.16	0.10	0.12	0.05	0.05	-0.08	0.26	0.14	-0.17	-0.19	0.45	-0.22	-0.17	0.29	-0.08
Stenotrophomonas	-0.22	0.04	-0.19	-0.33	0.11	-0.12	0.09	-0.02	0.02	-0.13	0.17	0.13	-0.19	-0.28	0.39	-0.19	-0.25	0.27	-0.15
Subdoligranulum	0.18	-0.12	0.07	-0.01	0.27	0.10	0.19	0.12	0.22	0.14	0.32	0.09	0.18	0.22	-0.05	0.27	0.12	0.29	0.18
Propionibacterium	-0.34	-0.25	-0.38	-0.46	0.00	0.03	0.04	0.01	-0.07	-0.24	0.17	-0.01	-0.33	-0.37	0.34	-0.07	-0.37	0.42	-0.26
Enhydrobacter	-0.02	0.16	-0.10	-0.26	0.09	-0.13	-0.09	0.04	0.16	0.04	0.25	0.29	0.00	-0.06	0.37	-0.18	-0.12	0.46	0.02
Acinetobacter	-0.41	-0.24	-0.46	-0.40	-0.14	-0.08	-0.08	-0.13	-0.24	-0.33	0.10	-0.08	-0.40	-0.44	0.25	0.02	-0.34	0.21	-0.35
Pseudomonas	-0.25	-0.08	-0.27	-0.34	0.03	-0.15	0.03	0.03	-0.05	-0.24	0.21	0.05	-0.19	-0.19	0.42	-0.05	-0.29	0.29	-0.16

$P < 0.05$ is marked with blue color, $P < 0.01$ is marked with red color.

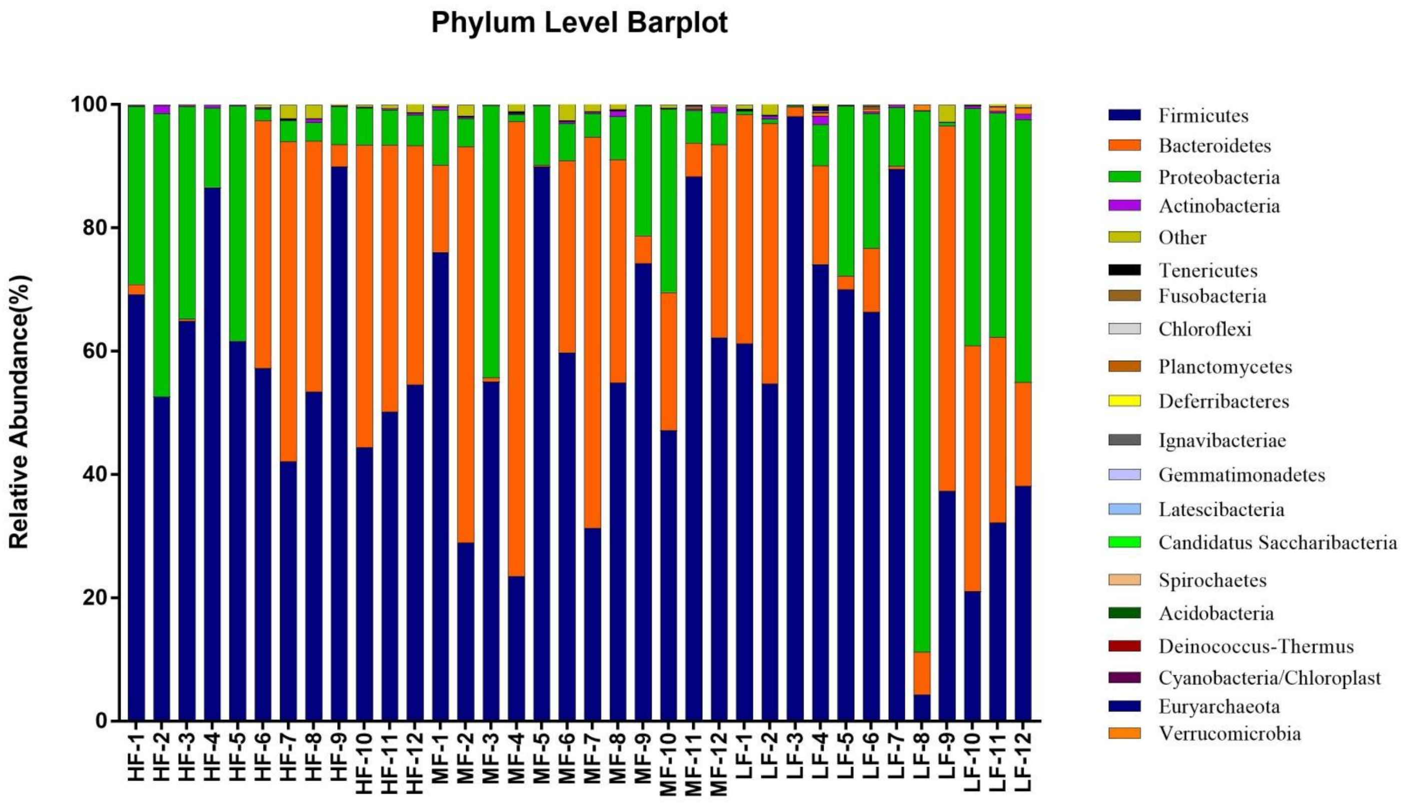

Figure S1: Taxonomy profile of microbiota composition at the phylum level.

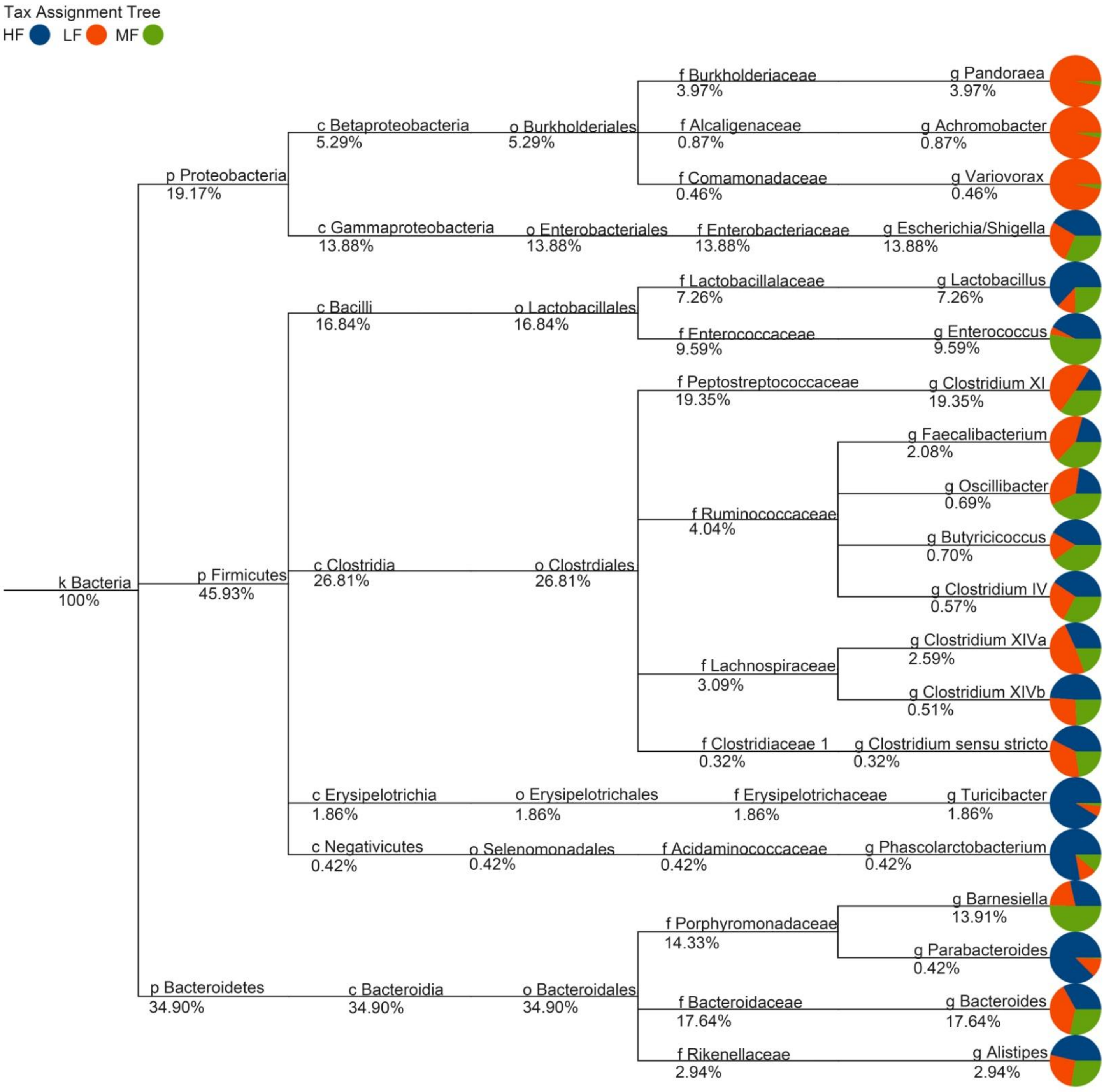

Figure S2: Taxonomy profile of microbiota composition at the genus level.
